# Resistance to Human-Reserved Antimicrobials in Clinical and Commensal *Enterococcus faecalis* and *Enterococcus faecium* from Dogs and Cats

**DOI:** 10.3390/antibiotics15080732

**Published:** 2026-07-29

**Authors:** Giulia Iamone, Alessandro Bellato, Ilaria Prandi, Maria Cristina Stella, Simona Paparone, Simona Zoppi, Cristina Marra, Renato Zanatta, Rosario Musumeci, Patrizia Robino, Patrizia Nebbia

**Affiliations:** 1 Department of Veterinary Sciences, University of Turin, Largo Paolo Braccini 2, 10095 Grugliasco, Italy; giulia.iamone@unito.it (G.I.); ilaria.prandi@unito.it (I.P.); mariacristina.stella@unito.it (M.C.S.); simona.paparone@unito.it (S.P.); cristina.marra@unito.it (C.M.); renato.zanatta@unito.it (R.Z.); patrizia.robino@unito.it (P.R.); patrizia.nebbia@unito.it (P.N.); 2 Istituto Zooprofilattico Sperimentale del Piemonte, Liguria e Valle d’Aosta Via Bologna 148, 10154 Torino, Italy; simona.zoppi@izsplv.it; 3 School of Medicine and Surgery, University of Milano-Bicocca, 20900 Monza, Italy; rosario.musumeci@unimib.it

**Keywords:** *Enterococcus faecalis*, *Enterococcus faecium*, dog, cat, antimicrobial resistance (AMR), MICs, human-reserved antimicrobials

## Abstract

**Background/Objectives:** Although companion animals are recognized as reservoirs of antimicrobial resistant enterococci, it remains unclear whether antimicrobial resistance differs between clinical and commensal isolates, particularly for antimicrobials reserved for human medicine. This study compared antimicrobial susceptibility profiles of clinical and commensal *E. faecalis* and *E. faecium* isolates from dogs and cats, focusing on antimicrobials reserved for human medicine under European regulation and EMA/AMEG classification. **Methods:** A total of 99 isolates (49 *E. faecalis* and 50 *E. faecium*) collected between 2022 and 2025 were analysed by broth microdilution, gradient diffusion, and disk diffusion methods. Antimicrobial resistance patterns were compared according to bacterial species and isolate origin. **Results:** *E. faecium* exhibited significantly higher resistance rates than *E. faecalis*, particularly to ampicillin, fluoroquinolones, erythromycin, nitrofurantoin, and vancomycin. Multidrug resistance was widespread, affecting all clinical *E. faecium* isolates and 72.2% of commensal *E. faecium*, whereas corresponding proportions in *E. faecalis* were 64.1% and 40.0%, respectively. Clinical *E. faecium* isolates showed significantly higher resistance than commensal isolates to antimicrobials commonly used in veterinary medicine, including ampicillin, fluoroquinolones, erythromycin, gentamicin, streptomycin, and tetracycline. Conversely, no significant differences were observed for antimicrobials reserved for human medicine, including linezolid, vancomycin, teicoplanin, tigecycline, daptomycin, rifampicin, and quinupristin/dalfopristin. **Conclusions:** The higher resistance observed in clinical isolates to veterinary antimicrobials is consistent with the selective pressure exerted by antimicrobial use in veterinary clinical settings, whereas resistance to human-reserved agents may reflect epidemiological processes that are independent of antimicrobial exposure in veterinary clinical settings. Continuous integrated One Health surveillance is essential to monitor the emergence and dissemination of these resistance phenotypes. The AMR profiles observed in companion animals further support the need for integrated One Health surveillance, combining veterinary, human, and environmental data to better understand the emergence and dissemination of resistant enterococci.

## 1. Introduction

Antimicrobial resistance (AMR) in *Enterococcus faecalis* and *Enterococcus faecium* represents a major global health threat, affecting human, animal, and environmental domains within a One Health framework. Enterococci are part of the gastrointestinal microbiota of both humans and animals, including companion animals such as dogs and cats, where they represent an important reservoir of antimicrobial resistance [1,2,3]. Simultaneously, they have emerged as opportunistic pathogens, responsible for a wide range of infections particularly in hospital settings [4,5]. In veterinary medicine, *Enterococcus* spp. are commonly associated with clinical conditions such as urinary tract infections, wound infections and, less commonly, bloodstream infections.

The dual role of enterococci as both commensals and opportunistic pathogens is especially relevant in companion animals, where close contact with humans may facilitate the exchange of resistant strains and resistance determinants. Faecal carriage in healthy dogs and cats contributes to the maintenance and dissemination of the community-acquired antibiotic resistance genes (ARGs), whereas clinical isolates reflect the selective pressure exerted by antimicrobial treatments in veterinary practice. This interplay underscores the importance of investigating AMR in both pathogenic and commensal isolates of humans and animals, within a unified One Health perspective [6,7].

Surveillance studies across Europe and North America evidenced that antimicrobial use in veterinary medicine has contributed to the selection of resistant enterococcal populations in companion animals [8,9,10]. In particular, resistance to several antimicrobial classes, including tetracyclines, macrolides, and aminoglycosides, is widespread among enterococci of veterinary origin. This is largely driven by mobile genetic elements (MGEs), such as plasmids, transposons, and integrative conjugative elements, which facilitate horizontal gene transfer and promote the dissemination of resistant enterococcal populations. Among the most frequently reported ARGs commonly associated with MGEs are *tet(M)*, *erm(B)*, *aac(6′)-Ie* and *aph(2″)-Ia* [11,12].

Particular concern is associated with resistance to antimicrobials reserved to human medicine exclusively. Several of these compounds were previously classified as Category A (“Avoid”) by the European Medicines Agency’s (EMA) Antimicrobial Advice Ad Hoc Expert Group [13] and are now included in the Human Reserved List established by Commission Implementing Regulation (EU) 2022/1255 [14], which legally prohibits their use in animals throughout the European Union. Among the non-authorized antimicrobials, several represent important therapeutic options for the treatment of severe infections caused by multidrug-resistant (MDR) Gram-positive bacteria in human medicine. Although resistance to these agents remains relatively uncommon among enterococci from companion animals, resistant isolates have been sporadically reported in different geographic regions [7,15]. These reports support the hypothesis that pets may contribute to the maintenance and dissemination of antimicrobial-resistant enterococci across different ecological compartments, raising concerns regarding the emergence of animal reservoirs of resistance to last-resort human antimicrobials.

Despite growing interest in enterococci in companion animals, studies on AMR across EMA antimicrobial categories remain limited, especially for comparative investigations evaluating differences between clinical and commensal isolates.

Therefore, this study aimed to determine whether AMR profiles differ between clinical and commensal *E. faecalis* and *E. faecium* isolates from dogs and cats, with particular emphasis on antimicrobials included in the EMA/AMEG classification (A–D) and those reserved exclusively for human medicine.

## 2. Results

### 2.1. Distribution of Isolates According to Bacterial Species, Host Species and Sample Type

A total of 99 isolates collected between 2022 and 2025 from dogs and cats were included in the study. Of these, 49 (49.5%) were identified as *E. faecalis* and 50 (50.5%) as *E. faecium*. The isolates originated from 69 dogs (69.7%), including 31 *E. faecalis* (44.9%) and 38 *E. faecium* (55.1%), and 30 cats (30.3%), including 18 *E. faecalis* (60.0%) and 12 *E. faecium* (40.0%). No significant association was observed between bacterial and host species (Odds Ratio = 1.8, 95% Confidence Interval = 0.7–4.9, *p* = 0.194).

Seventy-one isolates (71.7%) were recovered from animals presenting clinical infections, including 39 *E. faecalis* (54.9%) and 32 *E. faecium* (45.1%), whereas 28 isolates (28.3%) were obtained from faecal samples of clinically healthy animals and classified as commensal isolates, comprising 10 *E. faecalis* (35.7%) and 18 *E. faecium* (64.3%). No significant association was observed between bacterial species and isolate origin (OR = 2.2, 95%CI = 0.8–6.1, *p* = 0.118). Among clinical samples, urinary tract infections represented the most frequent source (47/71; 66.2%), followed by skin lesions and infected surgical wounds (24/71; 33.8%).

Detailed information on isolates’ origin, host species, and sample type is provided in Supplementary Table S1.

### 2.2. Antimicrobial Resistance Profiles

Since no statistically significant differences in AMR profiles were observed between isolates from dogs and cats, data from both animal species were pooled for subsequent analyses.

Phenotypic antimicrobial susceptibility testing revealed widespread resistance to several antimicrobial classes, with substantial differences between *E. faecalis* and *E. faecium*, and isolates’ origin (Table 1). Overall, *E. faecium* exhibited significantly higher resistance rates than *E. faecalis*, particularly against ampicillin (*p* < 0.001), erythromycin (*p* = 0.005), fluoroquinolones (all *p* < 0.001), nitrofurantoin (*p* < 0.001), and vancomycin (*p* = 0.001). No significant difference was observed for chloramphenicol (*p* = 0.158), gentamicin (*p* = 0.527), linezolid (*p* = 1.000), rifampicin (*p* = 0.250), streptomycin (*p* = 0.228), teicoplanin (*p* = 0.507), tetracycline (*p* = 0.965), and tigecycline (*p* = 1.000). No resistance was observed against daptomycin, while the two bacterial species could not be compared for imipenem and quinupristin/dalfopristin resistance due to intrinsic resistance.

The highest resistance rates were observed for fluoroquinolones, tetracycline, erythromycin, rifampicin, and in *E. faecium* for quinupristin/dalfopristin. Detailed resistance frequencies for all tested antimicrobials are reported in Table 1. Additional details are reported in Supplementary Table S2.

*E. faecium* consistently displayed broader resistance profiles than *E. faecalis*, particularly for β-lactams and fluoroquinolones.

### 2.3. Comparison Between Clinical and Commensal Isolates

Although clinical isolates generally exhibited a higher rate of resistance than commensal isolates, no significant differences were observed for *E. faecalis* (Supplementary Table S3). Conversely, in *E. faecium*, statistically significant differences between clinical and commensal isolates were observed for ampicillin (*p* < 0.001), ciprofloxacin (*p* < 0.001), erythromycin (*p* = 0.049), gentamicin (*p* = 0.046), streptomycin (*p* = 0.049), and tetracycline (*p* = 0.047) (Supplementary Table S4).

### 2.4. Multidrug Resistance and Resistance to Antimicrobials Classified as EMA/AMEG Category A or Reserved for Human Medicine

**Figure 1:**
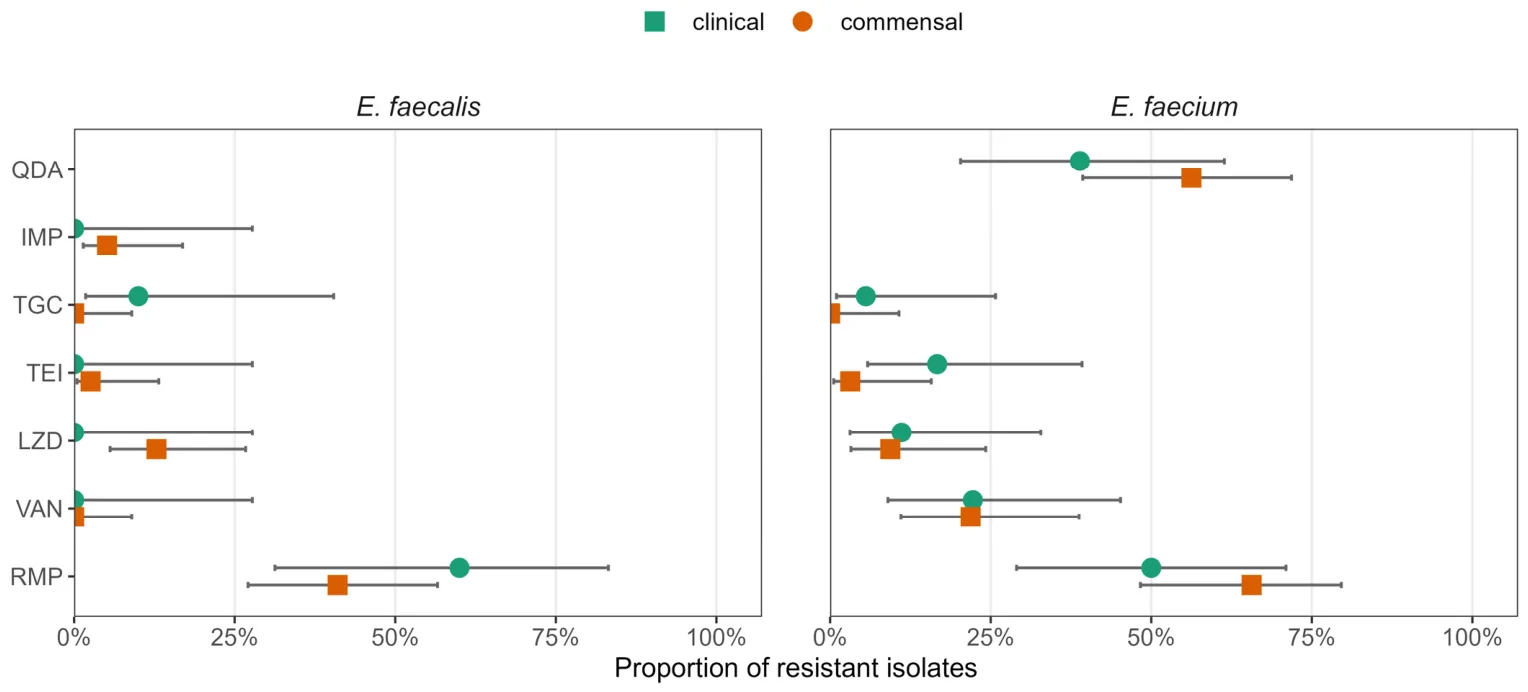
Proportion of isolates resistant to EMA/AMEG [13] Category A and EU human-reserved antimicrobials [14] among commensal and clinical *Enterococcus* isolates from dogs and cats. [DAP, daptomycin; IMP, imipenem; LZD, linezolid; QDA, quinupristin/dalfopristin; RMP, rifampicin; TEI, teicoplanin; TGC, tigecycline; VAN, vancomycin].

A high proportion of MDR enterococci was observed among the investigated isolates (Table 1). According to the international definition proposed by Magiorakos et al. (2012) [16], MDR was defined as resistance to at least three antimicrobial classes. Resistance rates to antimicrobials classified by the EMA/AMEG [13] as Category A (“Avoid”) and/or reserved for human medicine under Commission Implementing Regulation (EU) 2022/1255 [14] are reported in Figure 1.

Among *E. faecalis*, 64.1% (25/39) of clinical isolates were classified as MDR, compared with 40.0% (4/10) of commensal isolates. In contrast, all *E. faecium* clinical isolates exhibited MDR phenotypes (32/32), while among commensal strains multidrug resistance was observed in 72.2% (13/18) isolates.

Overall, *E. faecium* displayed the highest MDR burden (*p* < 0.001), combining resistance to β-lactams, fluoroquinolones, macrolides, tetracyclines, aminoglycosides and rifampicin. No isolates met the criteria for extensively drug-resistant (XDR) or pandrug-resistant (PDR) phenotypes. The clinical isolates of *E. faecium* showed MDR phenotype significantly more frequently than commensal isolates of the same species (*p* = 0.029).

Given their critical importance for human health, particular attention was devoted to antimicrobials classified within EMA/AMEG [13] Category A (“Avoid”) and to antimicrobial classes reserved exclusively for human use. Resistance to these compounds is of particular concern because they include some of the last therapeutic options available for the treatment of severe MDR infections in humans.

Among these antimicrobials, rifampicin and quinupristin–dalfopristin resistance represents the most striking findings (Figure 1). Rifampicin resistance was detected in 41% (16/39) of clinical *E. faecalis* isolates and 65.6% (21/32) of clinical *E. faecium* isolates. Among commensal isolates, resistance frequencies were 60% (6/10) and 50% (9/18), respectively. Quinupristin–dalfopristin-resistant strains were 56.3% (18/32) of *E. faecium* clinical isolates and 38.9% (7/18) of faecal isolates. Linezolid resistance was detected less frequently but remained epidemiologically relevant, as resistant isolates accounted for 12.8% (5/39) of clinical *E. faecalis* and 9.4% (3/32) of clinical *E. faecium* isolates. In addition, two commensal *E. faecium* isolates exhibited a resistant phenotype to linezolid.

Tigecycline maintained excellent in vitro activity against the investigated enterococcal population. Resistance was detected only sporadically, involving one commensal isolate of *E. faecalis* and one commensal isolate of *E. faecium*.

Vancomycin resistance was observed exclusively among *E. faecium* isolates, both clinical (7/32; 21.9%) and faecal (4/18; 22.2%), whereas teicoplanin and imipenem resistance was detected only sporadically. The simultaneous occurrence of widespread MDR phenotypes and resistance to antimicrobials classified in EMA/AMEG [13] Category A and/or reserved exclusively for human medicine under current European legislation highlights the potential role of companion animals as reservoirs of clinically relevant antimicrobial resistance within a One Health context.

## 3. Discussion

The present study provides an updated overview of antimicrobial resistance patterns in *E. faecalis* and *E. faecium* isolated from dogs and cats, highlighting the widespread occurrence of multidrug-resistant (MDR) enterococci in companion animals and the detection of resistance phenotypes toward antimicrobials reserved for human medicine. Overall, these findings indicate that companion animals may contribute to the maintenance of clinically relevant AMR determinants and highlight their potential relevance within the epidemiology of antimicrobial resistance.

As expected, *E. faecium* exhibited broader resistance profiles than *E. faecalis*, particularly against β-lactams, fluoroquinolones, rifampicin, and several critically important antimicrobials. High resistance frequencies were observed for fluoroquinolones, tetracycline, erythromycin, and rifampicin, with MDR phenotypes detected in nearly all *E. faecium* isolates and in most clinical *E. faecalis* isolates. Resistance to tetracyclines and macrolides, particularly erythromycin, represents one of the most recurrent findings in enterococci from companion animals, highlighting the selective pressure exerted by the extensive use of these antimicrobial classes in veterinary medicine [17]. Overall, these findings are consistent with previous veterinary and human studies showing the remarkable adaptive capacity of *E. faecium* under antimicrobial selective pressure [8,18].

Several studies conducted in dogs and cats have consistently reported high frequencies of MDR enterococci, particularly among *E. faecalis* and *E. faecium* isolates from both healthy and hospitalized animals [7,8,15].

However, substantial variability in MDR proportion has been observed among studies, depending on the host population, geographical setting, and origin of the isolates [8,19].

Recent evidence [20] suggests that multidrug-resistant enterococci carried by companion animals may also be disseminated into the environment through faecal shedding, supporting the role of pets as reservoirs of antimicrobial resistance within a One Health framework. Clinical isolates generally exhibited higher resistance rates than commensal isolates, consistent with previous studies indicating that antimicrobial exposure in veterinary clinical settings exerts selective pressure favouring the emergence and persistence of resistant enterococcal populations [1,8]. However, this pattern was not uniform across antimicrobial classes. In *E. faecium*, significant differences between clinical and commensal isolates were observed only for antimicrobials commonly used in veterinary practice, including ampicillin, fluoroquinolones, gentamicin, macrolides, and tetracyclines, supporting the role of antimicrobial exposure as a major driver of resistance selection in clinical settings. In contrast, no significant differences were detected for antimicrobials primarily reserved for human medicine. This finding suggests that resistance to these critically important compounds may not be primarily shaped by selective pressure within veterinary practice, but rather by alternative mechanisms, such as the circulation of resistant clones or mobile genetic elements carrying resistance determinants. No significant differences between clinical and commensal isolates were observed for any antimicrobial in *E. faecalis*, highlighting species-specific differences in the epidemiology of antimicrobial resistance.

One of the most relevant findings of this study was the detection of resistance phenotypes toward antimicrobials of critical importance for human medicine, including linezolid, quinupristin/dalfopristin, rifampicin, and vancomycin. Under Commission Implementing Regulation (EU) 2022/1255 [14], adopted according to the criteria established by Commission Delegated Regulation (EU) 2021/1760 [21], these antimicrobials are reserved exclusively for human use and are no longer authorized in veterinary medicine within the European Union. They represent last-resort therapeutic options for the treatment of severe infections caused by MDR bacteria. Therefore, the detection of resistant enterococci in companion animals is of particular One Health concern, as it may indicate the circulation of clinically relevant resistance determinants at the human–animal interface, with potential implications for public health. Rather than reflecting direct antimicrobial exposure in veterinary medicine, these resistance phenotypes are more likely to indicate the circulation of clinically relevant resistance determinants maintained through alternative ecological and epidemiological pathways.

Quinupristin/dalfopristin resistance was mainly observed in *E. faecium*, whereas *E. faecalis* was considered intrinsically resistant and was therefore not interpreted. The high frequency of resistance observed among our *E. faecium* isolates is noteworthy, particularly because streptogramins are not commonly used in companion-animal practice. Similar observations have been reported in human clinical *E. faecium* isolates, where reduced susceptibility to quinupristin/dalfopristin was detected even in the absence of previous exposure to the drug or veterinary use of virginiamycin, suggesting that additional mechanisms, co-selection, or clonal dissemination may contribute to this phenotype [22]. Moreover, previous studies have shown that resistance to quinupristin/dalfopristin may occur in *E. faecium* from humans, animals, and food products, supporting the hypothesis that streptogramin resistance determinants can circulate across different ecological compartments [23,24,25]. These observations suggest that the persistence of streptogramin resistance may depend more on co-selection and dissemination than on direct exposure. A similar epidemiological pattern was observed for linezolid. Linezolid was detected in both *E. faecalis* and *E. faecium* clinical isolates and in commensal strains of *E. faecalis*. Since linezolid is not authorized for veterinary use in the European Union, the occurrence of resistant isolates may suggest indirect acquisition of resistance determinants through horizontal gene transfer or environmental contamination. Previous studies [25] have demonstrated the circulation of linezolid resistance genes among enterococci isolated from animals, food, and humans, emphasizing the interconnectedness of antimicrobial resistance across different ecological compartments. Linezolid resistance was higher than generally reported in the literature, where resistance remains uncommon even among (VRE) isolates [18,26,27]. In fact, vancomycin resistance was restricted to *E. faecium* isolates, whereas all *E. faecalis* remained susceptible. This finding is consistent with the epidemiology of enterococci, in which acquired glycopeptide resistance is predominantly associated with *E. faecium* [28], confirming the well-recognized role of this species as the principal reservoir of VRE. However, the proportion of resistant isolates we observed exceeded that reported in comparable clinical studies, suggesting a higher local circulation of vancomycin-resistant *E. faecium* [18,26].

However, not all antimicrobials reserved for human medicine showed the same epidemiological pattern. Interestingly, tigecycline resistance was observed only among faecal isolates of both *E. faecalis* and *E. faecium*, while all clinical isolates remained susceptible. Although based on a small number of isolates, this distribution suggests that some tigecycline-resistant enterococci may persist within the intestinal microbiota of healthy companion animals. Moreover, Daptomycin resistance was not detected among either clinical or commensal enterococci. This finding is consistent with previous reports indicating that daptomycin resistance remains uncommon among *Enterococcus* spp., supporting the preserved activity of this last-resort antimicrobial [29].

The occurrence of clinically relevant resistance in companion animals probably reflects the complex interaction of several epidemiological pathways, including close human–animal contact, shared environments, and the food chain [7,20,25]. These interconnected routes may facilitate the persistence and dissemination of resistant enterococci and their resistance determinants across different ecological niches.

These findings have important implications for AMR surveillance and support the adoption of a One Health framework for future surveillance activities. Given their close and prolonged contact with humans, companion animals may contribute to the circulation of MDR enterococci within households, veterinary healthcare settings, and the environment. In this context, the detection of MDR isolates displaying resistance to last-resort antimicrobials reserved for human medicine is of particular concern, as it highlights the need for integrated AMR surveillance across the veterinary, human, and environmental sectors. Continuous monitoring of resistance to these critically important antimicrobials is essential to preserve the effectiveness of therapeutic options that represent some of the last available treatments for severe infections caused by MDR bacteria.

This study presents some limitations: the number of isolates was relatively limited and originated from a restricted geographical area, which may limit the generalizability of the findings; the present investigation was based exclusively on phenotypic antimicrobial susceptibility testing, and the genetic determinants underlying the observed resistance phenotypes were not investigated. Whole-genome sequencing is currently underway. It will enable the identification of AMR genes, mobile genetic elements, and clonal lineages, providing a more comprehensive understanding of the mechanisms driving AMR in these isolates. In addition, individual antimicrobial treatment histories were not available for the enrolled animals, precluding the assessment of associations between previous antimicrobial exposure and the observed resistance phenotypes.

Despite these limitations, the study provides important epidemiological data highlighting the importance of companion animals in the epidemiology of AMR and the need for integrated surveillance across the human, animal, and environmental sectors.

## 4. Materials and Methods

### 4.1. Sampling

Enterococcal isolates included in this study were prospectively collected between 2022 and 2025, corresponding to the duration of a research project funded by Fondazione CRT (grant ROL80626). Isolates were obtained from dogs and cats examined at the Veterinary Teaching Hospital of the University of Turin and from samples submitted to the Istituto Zooprofilattico Sperimentale del Piemonte, Liguria e Valle d’Aosta. A total of 99 isolates were analysed, including 50 *E. faecium* and 49 *E. faecalis*. Seventy-one samples consisted of clinical isolates, recovered from urine samples, skin lesions, or infected surgical wounds of animals with clinically diagnosed infections. The remaining 28 commensal isolates were obtained from non-invasively collected faecal samples of clinically healthy dogs and cats presented to the Veterinary Teaching Hospital for conditions unrelated to infectious diseases.

### 4.2. Microorganism Isolation and Identification

Biological samples (urine, swabs or faeces) were inoculated onto Columbia Blood Agar plates (Oxoid, Basingstoke, UK) and incubated at 37 °C for 24h. Single colonies presumably belonging to the genus *Enterococcus* were identified to species level with MALDI-TOF mass spectrometry (Bruker Daltonics GmBH, Bremen, Germany) following manufacturer recommendations. Only *E. faecalis* and *E. faecium* were retained and subcultured on Tryptic Soy Agar (TSA, Oxoid) plates. Species confirmation was conducted with 16S rRNA-ribotyping [30] after DNA extraction (InstaGene Matrix Kit, Bio-Rad, Hercules, CA, USA) according to the manufacturer’s recommendations, with minor modifications. Selected isolates were stored at −80 °C in Tryptic soy Broth (TSB; Oxoid) supplemented with 20% glycerol (Merk, Darmstadt, Germany) until further analysis.

### 4.3. Antimicrobial Susceptibility Testing

The antimicrobial agents included in the study and their classification according to the European Medicines Agency (EMA) Antimicrobial Advice Ad Hoc Expert Group (AMEG) categorization [13] and Commission Implementing Regulation (EU) 2022/1255 [14] are reported in Table 2.

MICs were determined using Sensititre EU Surveillance *Enterococcus* EUVENC plates (Thermo Fisher, Milan, Italy), according to the manufacturer’s instructions. The plates contained the following antimicrobial agents: ampicillin (AMP, 0.5–64 µg/mL), ciprofloxacin (CIP, 0.12–16 µg/mL), chloramphenicol (CHL, 4–128 µg/mL), vancomycin (VAN, 1–128 µg/mL), teicoplanin (TEI, 0.5–64 µg/mL), tetracycline (TET, 1–128 µg/mL), erythromycin (ERY, 1–128 µg/mL), tigecycline (TGC, 0.03–4 µg/mL), linezolid (LZD, 0.5–64 µg/mL), and gentamicin (GEN, 8–1024 µg/mL). Following incubation, MICs were read using the Vizion™ Digital MIC Viewing System (Sensititre™, Thermo Scientific, Milan, Italy). Daptomycin (DAP, 0.016–256 µg/mL) and quinupristin–dalfopristin (QDA, 0.002–32 µg/mL) susceptibility was determined separately using a gradient diffusion method (MIC test strip^®^, Liofilchem, Roseto degli Abruzzi, Italy).

The following antimicrobial agents (Oxoid) were evaluated through the disk diffusion test (DDT): rifampicin (RMP, 5 µg), marbofloxacin (MAR, 5 µg; Fatro S.p.A., Ozzano dell’Emilia, Italy), enrofloxacin (ENR, 5 µg), streptomycin (SP, 300 µg), imipenem (IMP, 10 µg), and nitrofurantoin (F, 100 µg). A 0.5 McFarland bacterial suspension was inoculated onto Mueller–Hinton agar (MHA, Oxoid) plates and, after incubation, inhibition zone diameters were measured. The disk diffusion test (DDT) was performed according to EUCAST 2025 guidelines [31].

### 4.4. AST Interpretation

Antimicrobial susceptibility results were interpreted according to the EUCAST Clinical Breakpoint Tables (2026) [31] for *Enterococcus* spp., when available (AMP, CIP, GEN, LZD, QDA, TEI, TGC, VAN, IMP and F). For antimicrobial agents not covered by EUCAST clinical breakpoints (CHL, ERY, TET, RMP, MAR, ENR and SP), CLSI criteria [32] were applied, whereas the EUCAST epidemiological cut-off value (ECOFF) was used for daptomycin (https://mic.eucast.org, last accessed on 10 June 2026). According to EUCAST definitions, isolates categorized as susceptible, increased exposure (I) were counted as susceptible in the statistical analyses.

### 4.5. Statistics

AST results were analysed with contingency tables and Fisher’s exact tests for testing independence of results. Only acquired resistance phenotypes were considered, whereas intrinsic resistance was excluded from comparisons between species and isolates’ origin. A *p*-value < 0.05 was considered indicative of statistical significance. To account for multiple testing and control the false discovery rate, *p*-values were corrected using the Benjamini–Hochberg method [33]. Data analysis was performed using R version 4.4.3 [34].

## 5. Conclusions

This study documents the widespread occurrence of multidrug-resistant *E. faecalis* and *E. faecium* in dogs and cats, with *E. faecium* exhibiting the highest resistance burden. More importantly, our findings indicate that antimicrobial resistance profiles differ according to the antimicrobial classes considered. Clinical isolates showed significantly higher resistance than commensal isolates only for antimicrobials commonly used in veterinary medicine, consistent with the selective pressure likely exerted by antimicrobial therapy. In contrast, no significant differences were observed for antimicrobials reserved for human medicine, suggesting that factors other than direct veterinary antimicrobial use may contribute to the resistance to these last-resort drugs.

These findings highlight the need to include both clinical and commensal enterococci in surveillance programs. Ongoing whole-genome sequencing will help elucidate the genetic basis of the observed resistance phenotypes and their dissemination. Overall, the detection of resistance to antimicrobials legally reserved for human medicine in both clinical and commensal isolates further supports the need for integrated surveillance across the human, animal, and environmental sectors to improve our understanding of the emergence and dissemination of these resistance phenotypes. The absence of an association between isolate origin and resistance to human-reserved antimicrobials, in contrast to the pattern observed for veterinary antimicrobials, suggests that different ecological and epidemiological processes may underlie these resistance patterns, providing a rationale for future investigations integrating phenotypic, genomic, and epidemiological data. The whole-genome sequencing analyses could represent the next step in testing these hypotheses by identifying the resistance determinants, mobile genetic elements, and clonal lineages underlying the observed phenotypic patterns. If confirmed, these findings should be evaluated in larger multicentre studies integrating veterinary, human, and environmental data.

## Figures and Tables

**Table 1:** Proportion of resistant isolates of *Enterococcus faecalis* and *Enterococcus faecium* isolated from clinical and faecal samples of dogs and cats.

Antimicrobials	*E. faecalis*		Subtotal	*E. faecium*		Subtotal
	Clinical samples (n = 39)	Faecal samples (n = 10)	49	Clinical samples (n = 32)	Faecal samples (n = 18)	50
Vancomycin	0 (0.0%)	0 (0.0%)	0 (0.0%)	7 (21.9%)	4 (22.2%)	11 (22.0%)
Teicoplanin	1 (2.6%)	0 (0.0%)	1 (2.0%)	1 (3.1%)	3 (16.7%)	4 (8.0%)
Tigecycline	0 (0.0%)	1 (10.0%)	1 (2.0%)	0 (0%)	1 (5.6%)	1 (2.0%)
Rifampicin	16 (41.0%)	6 (60.0%)	22 (44.9%)	21 (65.6%)	9 (50.0%)	30 (60.0%)
Quinupristin– dalfopristin	IR	IR	IR	18 (56.3%)	7 (38.9%)	25 (50.0%)
Imipenem	2 (5.1%)	0 (0.0%)	2 (4.0%)	IR	IR	IR
Daptomycin	0 (0.0%)	0 (0.0%)	0 (0.0%)	0 (0.0%)	0 (0.0%)	0 (0.0%)
Linezolid	5 (12.8%)	0 (0.0%)	5 (10.2%)	3 (9.4%)	2 (11.1%)	5 (10.0%)
Ciprofloxacin	8 (20.5%)	0 (0.0%)	8 (16.3%)	29 (90.6%)	4 (22.2%)	33 (66.0%)
Marbofloxacin	14 (35.9%)	3 (30.0%)	17 (34.7%)	30 (93.8%)	13 (72.2%)	43 (86.0%)
Enrofloxacin	22 (56.4%)	6 (60.0%)	28 (57.1%)	32 (100.0%)	15 (83.3%)	47 (94.0%)
Gentamicin	4 (10.3%)	2 (20.0%)	6 (12.2%)	10 (31.3%)	0 (0.0%)	10 (20.0%)
Streptomycin	14 (35.9%)	5 (50.0%)	19 (38.8%)	21 (65.6%)	5 (27.8%)	26 (52.0%)
Ampicillin	4 (10.2%)	0 (0.0%)	4 (8.2%)	29 (90.6%)	4 (22.2%)	33 (66.0%)
Erythromycin	25 (64.1%)	5 (50.0%)	30 (61.2%)	31 (96.9%)	13 (72.2%)	44 (88.0%)
Nitrofurantoin	0 (0.0%)	0 (0.0%)	0 (0.0%)	14 (43.8%)	4 (22.2%)	18 (36.0%)
Chloramphenicol	8 (20.5%)	2 (20.0%)	9 (20.4%)	4 (12.5%)	0 (0.0%)	4 (8.0%)
Tetracycline	29 (74.4%)	6 (60.0%)	35 (71.4%)	26 (81.3%)	8 (44.4%)	34 (68%)
MDR	25 (64.1%)	4 (40.0%)	29 (59.2%)	32 (100.0%)	13 (72.2%)	45 (90.0%)

IR: intrinsic resistance. MDR: multi-drug resistant.

**Table 2:** Classification of the antimicrobial agents included in the study according to the European Medicines Agency’s Antimicrobial Advice Ad Hoc Expert Group (AMEG) categorization and Regulation (EU) 2022/1255.

Antimicrobial Class	Antimicrobial Agent	AMEG Category	EU 2022/1255 (Human-Only) *
Carbapenem	Imipenem *	-	yes
Glycilcycline	Tigecycline *	-	yes
Glycopeptide	Vancomycin *	-	yes
Glycopeptide	Teicoplanin *	-	yes
Lipopeptide	Daptomycin *	-	yes
Oxazolidinone	Linezolid *	-	yes
Rifamycin	Rifampicin	A—Avoid	
Streptogramin combination	Quinupristin–dalfopristin	A—Avoid	
Fluoroquinolone	Ciprofloxacin	B—Restrict	
	Enrofloxacin	B—Restrict	
	Marbofloxacin	B—Restrict	
Aminoglycoside	Gentamicin	C—Caution	
	Streptomycin	C—Caution	
Aminopenicillin (β-lactam)	Ampicillin	C—Caution	
Macrolide	Erythromycin	C—Caution	
Phenicol	Chloramphenicol	C—Caution	
Tetracycline	Tetracycline	D—Prudence	
	Nitrofurantoin	Not categorized	

* Antimicrobials legally reserved for human medicine under Regulation (EU) 2022/1255 [14]. These antibiotics were included exclusively for antimicrobial resistance surveillance purposes.

## Data Availability

The datasets generated and/or analysed during the current study are available from the corresponding author upon reasonable request.

## Supplementary Materials

The following supporting information can be downloaded at: https://www.mdpi.com/article/10.3390/antibiotics15080732/s1. Table S1: Detailed information on host species, sample type and isolated species. Table S2: Counts (proportion) of *Enterococcus faecalis* and *Enterococcus faecium* isolates resistant to each tested antibiotic and multi-drug resistant (MDR) isolates. The table shows the difference between proportions of resistant *E. faecium* and *E. faecalis*, the odds ratio as measure of association (with 95% confidence interval) and the *p*-value for each comparison. Table S3: Counts (proportion) of clinical and commensal isolates of *Enterococcus faecalis* resistant to each tested antibiotic and multi-drug resistant (MDR) isolates. The table shows the difference between proportions of resistant clinical and commensal isolates, the odds ratio as measure of association (with 95% confidence interval) and the *p*-value for each comparison. Table S4: Counts (proportion) of clinical and commensal isolates of *Enterococcus faecium* resistant to each tested antibiotic and multi-drug resistant (MDR) isolates. The table shows the difference between proportions of resistant clinical and commensal isolates, the odds ratio as measure of association (with 95% confidence interval) and the *p*-value for each comparison.

## Abbreviations

The following abbreviations are used in this manuscript:

EMA	European Medicines Agency
AMEG	Antimicrobial Advice Ad Hoc Expert Group
AMR	Antimicrobial resistance
MIC	Minimum inhibitory concentration
ARG	Antimicrobial resistance genes
MGE	Mobile genetic elements
MDR	Multi-drug resistant
OR	Odds ratio
95%CI	95% Confidence Interval
IR	Intrinsic resistance
XDR	Extensive-drug resistant
PDR	Pan-drug resistant
DAP	Daptomycin
IMP	Imipenem
LZD	Linezolid
QDA	Quinupristin/dalfopristin
RMP	Rifampicin
TEI	Teicoplanin
TGC	Tigecycline
VAN	Vancomycin
VRE	Vancomycin-resistant enterococci
MALDI-TOF	Matrix-assisted laser desorption/ionization—time of flight
TSA	Tryptic soy agar
TSB	Tryptic soy broth
AMP	Ampicillin
CHL	Chloramphenicol
TET	Tetracycline
ERY	Erythromycin
GEN	Gentamicin
CIP	Ciprofloxacin
ENR	Enrofloxacin
MAR	Marbofloxacin
DDT	Disk diffusion test
SP	Streptomycin
MHA	Mueller–Hinton agar
EUCAST	European Committee for Antimicrobial Susceptibility Testing
CLSI	Clinical and Laboratory Standard Institute
ECOFF	Epidemiological cut-off

## References

[1] Stępień-Pyśniak D., Bertelloni F., Dec M., Cagnoli G., Pietras-Ożga D., Urban-Chmiel R., Ebani V.V. (2021). Characterization and comparison of Enterococcus spp. isolates from feces of healthy dogs and urine of dogs with UTIs. Animals.

[2] Scarpellini R., Giunti M., Pontiero A., Savini F., Esposito E., Piva S. (2023). Two cases of bloodstream infections associated with opportunistic bacterial species (Enterococcus hirae and Enterobacter xiangfangensis) in companion animals. BMC Vet. Res..

[3] Danieli D., Amadori M., Crimi S., Pregnolato F., Caruso C., Gambino G., Re G., Vercelli C. (2026). Antimicrobial resistant isolates in surgical and bite wounds in dogs and cats: A 12-year retrospective analysis. Animals.

[4] Arias C.A., Murray B.E. (2012). The rise of the Enterococcus: Beyond vancomycin resistance. Nat. Rev. Microbiol..

[5] O’Driscoll T., Crank C.W. (2015). Vancomycin-resistant enterococcal infections: Epidemiology, clinical manifestations, and optimal management. Infect. Drug Resist..

[6] Iseppi R., Messi P., Anacarso I., Bondi M., Sabia C., Condò C., de Niederhausern S. (2015). Antimicrobial resistance and virulence traits in Enterococcus strains isolated from dogs and cats. New Microbiol..

[7] Moon B.-Y., Ali M.S., Choi J.-H., Heo Y.-E., Lee Y.-H., Kang H.-S., Kim T.-S., Yoon S.-S., Moon D.-C., Lim S.-K. (2023). Antimicrobial resistance profiles of *Enterococcus faecium* and *Enterococcus faecalis* isolated from healthy dogs and cats in South Korea. Microorganisms.

[8] Osman M., Altier C., Cazer C. (2023). Antimicrobial resistance among canine enterococci in the northeastern United States, 2007–2020. Front. Microbiol..

[9] Tumpa A., Štritof Z., Pintarić S. (2022). Prevalence and antimicrobial susceptibility of Enterococcus spp. from urine of dogs and cats in northwestern Croatia. Res. Vet. Sci..

[10] van den Bunt G., Top J., Hordijk J., de Greeff S.C., Mughini-Gras L., Corander J., van Pelt W., Bonten M.J.M., Fluit A.C., Willems R.J.L. (2018). Intestinal carriage of ampicillin- and vancomycin-resistant *Enterococcus faecium* in humans, dogs and cats in The Netherlands. J. Antimicrob. Chemother..

[11] Hegstad K., Mikalsen T., Coque T.M., Werner G., Sundsfjord A. (2010). Mobile genetic elements and their contribution to the emergence of antimicrobial-resistant *Enterococcus faecalis* and *Enterococcus faecium*. Clin. Microbiol. Infect..

[12] Palmer K.L., Kos V.N., Gilmore M.S. (2010). Horizontal gene transfer and the genomics of enterococcal antibiotic resistance. Curr. Opin. Microbiol..

[13] (2020). Categorisation of Antibiotics Used in Animals Promotes Responsible Use to Protect Public Health.

[14] European Commission (2022). Commission Implementing Regulation (EU) 2022/1255 of 19 July 2022 Designating Antimicrobials or Groups of Antimicrobials Reserved for the Treatment of Certain Infections in Humans in Accordance with Regulation (EU) 2019/6 of the European Parliament and of the Council. Off. J. Eur. Union.

[15] Ben Said L., Dziri R., Sassi N., Lozano C., Ben Slama K., Ouzari I., Torres C., Klibi N. (2017). Species distribution, antibiotic resistance and virulence traits in canine and feline enterococci in Tunisia. Acta Vet. Hung..

[16] Magiorakos A.-P., Srinivasan A., Carey R.B., Carmeli Y., Falagas M.E., Giske C.G., Harbarth S., Hindler J.F., Kahlmeter G., Olsson-Liljequist B. (2012). Multidrug-resistant, extensively drug-resistant and pandrug-resistant bacteria: An international expert proposal for interim standard definitions for acquired resistance. Clin. Microbiol. Infect..

[17] Torres C., Alonso C.A., Ruiz-Ripa L., León-Sampedro R., Del Campo R., Coque T.M. (2018). Antimicrobial Resistance in Enterococcus spp. of Animal Origin. Microbiol. Spectr..

[18] Sannathimmappa M.B., Nambiar V., Aravindakshan R., Al-Risi E.S. (2023). Clinical Profile and Antibiotic Susceptibility Pattern of *Enterococcus faecalis* and *Enterococcus faecium* with an Emphasis on Vancomycin Resistance. Biomed. Biotechnol. Res. J..

[19] Oguttu J.W., Qekwana D.N., Odoi A. (2021). Prevalence and Predictors of Antimicrobial Resistance Among *Enterococcus* spp. from Dogs Presented at a Veterinary Teaching Hospital, South Africa. Front. Vet. Sci..

[20] Ribeiro J., Lameiras R., Silva V., Igrejas G., Cortez Nunes F., Ribeiro A.I., Mateus T.L., Poeta P. (2026). Occurrence and characterization of antimicrobial-resistant and virulent Enterococcus spp. in dog feces from urban green spaces in Porto (Portugal). Antibiotics.

[21] European Commission (2021). Commission Delegated Regulation (EU) 2021/1760 of 26 May 2021 supplementing Regulation (EU) 2019/6 of the European Parliament and of the Council by establishing the criteria for the designation of antimicrobials to be reserved for the treatment of certain infections in humans. Off. J. Eur. Union..

[22] Karanika M., Prati A., Kiritsi M., Spiliopoulou I., Neonakis I., Anifantaki M., Petinaki E. (2008). Reduced susceptibility to quinupristin/dalfopristin in *Enterococcus faecium* in Greece without prior exposure to the agent. Int. J. Antimicrob. Agents.

[23] Soltani M., Beighton D., Philpott-Howard J., Woodford N. (2000). Mechanisms of resistance to quinupristin-dalfopristin among isolates of *Enterococcus faecium* from animals, raw meat, and hospital patients in Western Europe. Antimicrob. Agents Chemother..

[24] Donabedian S.M., Perri M.B., Vager D., Hershberger E., Malani P., Simjee S., Chow J., Vergis E.N., Muder R.R., Gay K. (2006). Quinupristin–Dalfopristin Resistance in *Enterococcus faecium* Isolates from Humans, Farm Animals, and Grocery Store Meat in the United States. J. Clin. Microbiol..

[25] Freitas A.R., Finisterra L., Tedim A.P., Duarte B., Novais C., Peixe L., ESCMID Study Group on Food- and Water-borne Infections (EFWISG) (2021). Linezolid- and Multidrug-Resistant Enterococci in Raw Commercial Dog Food, Europe, 2019–2020. Emerg. Infect. Dis..

[26] Ayobami O., Willrich N., Reuss A., Eckmanns T., Markwart R. (2020). The ongoing challenge of vancomycin-resistant *Enterococcus faecium* and *Enterococcus faecalis* in Europe: An epidemiological analysis of bloodstream infections. Emerg. Microbes Infect..

[27] Peykov S., Kirov B., Strateva T. (2025). Linezolid in the Focus of Antimicrobial Resistance of Enterococcus Species: A Global Overview of Genomic Studies. Int. J. Mol. Sci..

[28] Lee T., Pang S., Abraham S., Coombs G.W. (2019). Antimicrobial-resistant CC17 *Enterococcus faecium*: The past, the present and the future. J. Glob. Antimicrob. Resist..

[29] Nair-Collins S., Godart G., Patel N., Yadav V., Larimore K., LeGout J.D., Chitale R., Durvasula R., Oring J. (2026). Understanding Daptomycin Resistance Mechanisms and Treatment Challenges in *Enterococcus faecium* Infection: A Case Series. Antibiotics.

[30] Fontana C., Favaro M., Pelliccioni M., Pistoia E.S., Favalli C. (2005). Use of the MicroSeq 500 16S rRNA gene-based sequencing for identification of bacterial isolates that commercial automated systems failed to identify correctly. J. Clin. Microbiol..

[31] European Committee on Antimicrobial Susceptibility Testing (EUCAST) (2025). Disk Diffusion Method for Antimicrobial Susceptibility Testing. https://www.eucast.org.

[32] CLSI (2023). Performance Standards for Antimicrobial Susceptibility Testing.

[33] Benjamini Y., Hochberg Y. (1995). Controlling the False Discovery Rate: A Practical and Powerful Approach to Multiple Testing. J. R. Stat. Soc. Ser. B Methodol..

[34] R Core Team (2025). R: A Language and Environment for Statistical Computing.

