# Supplementary material for: Resistance to Human-Reserved Antimicrobials in Clinical and Commensal Enterococcus faecalis and Enterococcus faecium from Dogs and Cats
Source: Antibiotics (Basel). 2026 Jul 29;15(8):732. doi: 10.3390/antibiotics15080732 (PMC13510246; doi:10.3390/antibiotics15080732)
Supplement: Supplementary file 1 [file antibiotics-15-00732-s001.zip › antibiotics-4461049-supplementary.pdf]

Supplementary Table S1. Detailed information on host species, sample type and isolated species.

ID	PET	LOCATION	SPECIES
01	dog	surgical wound	*E. faecalis*
02	dog	surgical wound	*E. faecalis*
03	dog	urine	*E. faecalis*
04	dog	cutaneous swab	*E. faecalis*
05	dog	surgical wound	*E. faecalis*
06	dog	surgical wound	*E. faecalis*
07	cat	urine	*E. faecalis*
08	dog	surgical wound	*E. faecalis*
09	dog	surgical wound	*E. faecalis*
10	dog	surgical wound	*E. faecalis*
11	cat	urine	*E. faecalis*
12	dog	surgical wound	*E. faecalis*
13	cat	surgical wound	*E. faecalis*
14	cat	ear swab	*E. faecalis*
15	cat	cutaneous swab	*E. faecalis*
16	cat	urine	*E. faecalis*
17	cat	urine	*E. faecalis*
18	cat	urine	*E. faecalis*
19	cat	urine	*E. faecalis*
20	dog	urine	*E. faecalis*
21	dog	nasal swab	*E. faecalis*
22	dog	ear swab	*E. faecalis*
23	dog	urine	*E. faecalis*
24	dog	urine	*E. faecalis*
25	dog	urine	*E. faecalis*
26	dog	urine	*E. faecalis*
27	dog	urine	*E. faecalis*
28	dog	urine	*E. faecalis*
29	dog	urine	*E. faecalis*
30	dog	urine	*E. faecalis*
31	cat	urine	*E. faecalis*
32	cat	urine	*E. faecalis*
33	cat	urine	*E. faecalis*
34	cat	urine	*E. faecalis*
35	cat	urine	*E. faecalis*
36	cat	urine	*E. faecalis*
37	cat	urine	*E. faecalis*
38	cat	urine	*E. faecalis*
39	cat	urine	*E. faecalis*
40	dog	surgical wound	*E. faecium*
41	dog	surgical wound	*E. faecium*
42	dog	surgical wound	*E. faecium*
43	dog	surgical wound	*E. faecium*
44	dog	surgical wound	*E. faecium*
45	dog	urine	*E. faecium*
46	dog	surgical wound	*E. faecium*
47	dog	urine	*E. faecium*
48	dog	urine	*E. faecium*
49	dog	urine	*E. faecium*
50	dog	urine	*E. faecium*
51	cat	urine	*E. faecium*
52	cat	radius	*E. faecium*
53	dog	urine	*E. faecium*
54	cat	urine	*E. faecium*
55	cat	urine	*E. faecium*
56	cat	urine	*E. faecium*
57	dog	abscess	*E. faecium*
58	cat	urine	*E. faecium*
59	cat	urine	*E. faecium*
60	dog	urine	*E. faecium*
61	cat	abdominal effusion	*E. faecium*
62	dog	surgical wound	*E. faecium*
63	cat	urine	*E. faecium*
64	dog	urine	*E. faecium*
65	dog	urine	*E. faecium*
66	dog	urine	*E. faecium*
67	dog	urine	*E. faecium*
68	dog	urine	*E. faecium*
69	cat	urine	*E. faecium*
70	cat	urine	*E. faecium*
71	cat	urine	*E. faecium*
72	dog	faeces	*E. faecalis*
73	dog	faeces	*E. faecalis*
74	dog	faeces	*E. faecalis*
75	dog	faeces	*E. faecalis*
76	dog	faeces	*E. faecalis*
77	dog	faeces	*E. faecalis*
78	dog	faeces	*E. faecalis*
79	dog	faeces	*E. faecalis*
80	dog	faeces	*E. faecalis*
81	dog	faeces	*E. faecalis*
82	dog	faeces	*E. faecium*
83	dog	faeces	*E. faecium*
84	dog	faeces	*E. faecium*
85	dog	faeces	*E. faecium*
86	dog	faeces	*E. faecium*
87	dog	faeces	*E. faecium*
88	dog	faeces	*E. faecium*
89	dog	faeces	*E. faecium*
90	dog	faeces	*E. faecium*
91	dog	faeces	*E. faecium*
92	dog	faeces	*E. faecium*
93	dog	faeces	*E. faecium*
94	dog	faeces	*E. faecium*
95	dog	faeces	*E. faecium*
96	dog	faeces	*E. faecium*
97	dog	faeces	*E. faecium*
98	dog	faeces	*E. faecium*
99	dog	faeces	*E. faecium*

Supplementary Table S2. Counts (proportion) of *Enterococcus faecalis* and *Enterococcus faecium* isolates resistant to each tested antibiotic and multi-drug resistant (MDR) isolates. The table shows the difference between proportions of resistant *E. faecium* and *E. faecalis*, the odds ratio as measure of association (with 95% confidence interval) and the p-value for each comparison.

Antimicrobials	*E. faecium* n = 50	*E. faecalis* n = 49	Difference	OR (95%CI)	Unadjusted p
Vancomycin	11 (22.0%)	0 (0.0%)	+22.0%	Inf. (2.9-Inf.)	< 0.001
Teicoplanin	4 (8.0%)	1 (2.6%)	+5.4%	4.1 (0.4-209.5)	0.362
Tigecycline	1 (2.0%)	1 (2.6%)	-0.6%	1.0 (0.0-78.4)	1.000
Rifampicin	30 (60.0%)	22 (44.9%)	+15.1%	1.8 (0.8-4.4)	0.161
Quinupristin–dalfopristin	25 (50.0%)	IR	-	-	-
Imipenem	IR	2 (4.0%)	-	-	-
Daptomycin	0 (0.0%)	0 (0.0%)	0.0%	-	-
Linezolid	5 (10.0%)	5 (10.2%)	-0.2%	1.0 (0.2-4.6)	1.000
Ciprofloxacin	33 (66.0%)	8 (16.3%)	+49.7%	.7 (3.5-29.6)	< 0.001
Marbofloxacin	43 (86.0%)	17 (34.7%)	+51.3%	11.2 (3.9-36.2)	< 0.001
Enrofloxacin	47 (94.0%)	28 (57.1%)	+36.9	11.5 (3.0-65.5)	< 0.001
Gentamicin	10 (20.0%)	6 (12.2%)	+7.8%	1.8 (0.5-6.5)	0.414
Streptomycin	26 (52.0%)	19 (38.8%)	+13.2%	1.7 (0.7-4.1)	0.228
Ampicillin	33 (66.0%)	4 (8.2%)	+57.8%	21.0 (6.2-94.0)	< 0.001
Erythromycin	44 (88.0%)	30 (61.2%)	+26.8%	4.6 (1.5-15.7)	0.002
Nitrofurantoin	18 (36.0%)	0 (0.0%)	+36.0%	Inf. (6.1-Inf.)	< 0.001
Chloramphenicol	4 (8.0%)	10 (20.4%)	-12.4%	0.3 (0.1-1.3)	0.091
Tetracycline	34 (68.0%)	35 (71.4%)	-3.4%	0.9 (0.3-2.2)	0.828
MDR	45 (90.0%)	29 (59.2%)	+30.8%	6.1 (1.9-23.1)	< 0.001

OR: Odds ratio; 95%CI: 95% Confidence interval.

Supplementary Table S3. Counts (proportion) of clinical and commensal isolates of *Enterococcus faecalis* resistant to each tested antibiotic and multi-drug resistant (MDR) isolates. The table shows the difference between proportions of resistant clinical and commensal isolates, the odds ratio as measure of association (with 95% confidence interval) and the p-value for each comparison.

Antimicrobials	Clinical n = 39	Commensal n = 10	Difference	OR (95%CI)	Unadjusted p
Vancomycin	0 (0.0%)	0 (0.0%)	0.0%	-	-
Teicoplanin	1 (2.6%)	0 (0.0%)	+2.6%	Inf. (0.0-Inf.)	1.000
Tigecycline	0 (0.0%)	1 (10.0%)	-10.0%	0.0 (0.0-10.0)	0.204
Rifampicin	16 (41.0%)	6 (60.0%)	-19.0%	0.5 (0.1-2.4)	0.311
Imipenem	2 (5.1%)	0 (0.0%)	+5.1%	Inf. (0.0-Inf.)	1.000
Daptomycin	0 (0.0%)	0 (0.0%)	0.0%	-	-
Linezolid	5 (12.8%)	0 (0.0%)	+12.8%	Inf. (0.2-Inf.)	0.569
Ciprofloxacin	8 (20.5%)	0 (0.0%)	+20.5%	Inf. (0.4-Inf.)	0.180
Marbofloxacin	14 (35.9%)	3 (30.0%)	+5.9%	1.3 (0.2-9.0)	1.000
Enrofloxacin	22 (56.4%)	6 (60.0%)	-3.6%	0.9 (0.2-4.3)	1.000
Gentamicin	4 (10.3%)	2 (20.0%)	-9.7%	0.5 (0.1-6.0)	0.588
Streptomycin	14 (35.9%)	5 (50.0%)	-14.1%	0.6 (0.1-2.9)	0.480
Ampicillin	4 (10.3%)	0 (0.0%)	+10.3%	Inf. (0.2-Inf.)	0.569
Erythromycin	25 (64.1%)	5 (50.0%)	+14.1%	1.8 (0.3-9.2)	0.480
Nitrofurantoin	0 (0.0%)	0 (0.0%)	0.0%	-	-
Chloramphenicol	8 (20.5%)	2 (20.0%)	+0.5%	1.0 (0.2-11.8)	1.000
Tetracycline	29 (74.4%)	6 (61.0%)	+14.4%	1.9 (0.3-10.1)	0.442
MDR	25 (64.1%)	4 (40.0%)	+24.1%	2.6 (0.5-14.9)	0.279

OR: Odds ratio; 95%CI: 95% Confidence interval; MDR: Multi-drug resistant.

Supplementary Table S4. Counts (proportion) of clinical and commensal isolates of *Enterococcus faecium* resistant to each tested antibiotic and multi-drug resistant (MDR) isolates. The table shows the difference between proportions of resistant clinical and commensal isolates, the odds ratio as measure of association (with 95% confidence interval) and the p-value for each comparison.

Antimicrobials	Clinical n = 32	Commensal n = 18	Difference	OR (95%CI)	Unadjusted p
Vancomycin	7 (21.9%)	4 (22.2%)	-0.3%	1.0 (0.2-5.4)	1.000
Teicoplanin	1 (3.1%)	3 (16.7%)	-13.6%	0.2 (0.0-2.3)	0.127
Tigecycline	0 (0.0%)	1 (5.6%)	-5.6%	0.0 (0.0.-21.9)	0.360
Rifampicin	21 (65.6%)	9 (50.0%)	+15.6%	1.9 (0.5-7.2)	0.370
Quinupristin–dalfopristin	18 (56.3%)	7 (38.9%)	+17.4%	2.0 (0.5-7.8)	0.377
Daptomycin	0 (0.0%)	0 (0.0%)	0.0%	-	-
Linezolid	3 (9.4%)	2 (11.1%)	-1.7%	0.8 (0.1-10.9)	1.000
Ciprofloxacin	29 (90.6%)	4 (22.2%)	+68.4%	30.1 (5.5-242.5)	< 0.001
Marbofloxacin	30 (93.8%)	13 (72.2%)	+21.6%	5.5 (0.8-65.5)	0.083
Enrofloxacin	32 (100.0%)	15 (83.3%)	+16.7%	Inf. (0.8-Inf.)	0.0416
Gentamicin	10 (31.3%)	0 (0.0%)	+31.3%	Inf. (1.5-Inf.)	0.009
Streptomycin	21 (65.6%)	5 (27.8%)	+37.8%	4.8 (1.2-22.0)	0.018
Ampicillin	29 (90.6%)	4 (22.2%)	+68.4%	30.1 (5.5-242.5)	< 0.001
Erythromycin	31 (96.9%)	13 (72.2%)	+24.7%	11.3 (1.1-579.5)	0.018
Nitrofurantoin	14 (43.8%)	4 (22.2%)	+21.6%	2.7 (0.6-13.7)	0.219
Chloramphenicol	4 (12.5%)	0 (0.0%)	+12.5%	Inf. (0.4-Inf.)	0.283
Tetracycline	26 (81.3%)	8 (44.4%)	+36.9%	5.2 (1.3-23.9)	0.012
MDR	32 (100.0%)	13 (72.2%)	+27.8%	Inf. (1.9-Inf.)	0.004

OR: Odds ratio; 95%CI: 95% Confidence interval; MDR: Multi-drug resistant.
